# The antipyretic efficacy and safety of propacetamol compared with dexibuprofen in febrile children: a multicenter, randomized, double-blind, comparative, phase 3 clinical trial

**DOI:** 10.1186/s12887-018-1166-z

**Published:** 2018-06-23

**Authors:** Seung Jun Choi, Sena Moon, Ui Yoon Choi, Yoon Hong Chun, Jung Hyun Lee, Jung Woo Rhim, Jin Lee, Hwang Min Kim, Dae Chul Jeong

**Affiliations:** 1 Department of Pediatrics, Asan Medical Center Children’s Hospital, University of Ulsan College of Medicine, Seoul, Republic of Korea; 2 Graduate School of Medicine, The Catholic University of Korea, College of Medicine, Seoul, Republic of Korea; 3 Department of Pediatrics, College of Medicine, The Catholic University of Korea, 222, Banpodaero, Seocho-gu, Seoul, 06591 Republic of Korea; 4 Department of Pediatrics, Hanjin General Hospital, Seoul, Republic of Korea; 5 Department of Pediatrics, Yonsei Christian Hospital, Wonju, Republic of Korea; 6 Vaccine Bio-research Institute, College of Medicine, The Catholic University of Korea, Seoul, Republic of Korea

**Keywords:** Children, Dexibuprofen, Fever, Propacetamol, Upper respiratory tract infection

## Abstract

**Background:**

We aimed to compare the antipyretic efficacy, safety, and tolerability between oral dexibuprofen and intravenous propacetamol in children with upper respiratory tract infection (URTI) presenting with fever.

**Methods:**

Patients aging from 6 months to 14 years admitted for URTI with axillary body temperature ≥ 38.0 °C were enrolled and randomized into the study or control group. Patients in the study group were intravenously infused with propacetamol and subsequently oral placebo medication was administered. Patients in the control group were intravenously infused with 100 mL of 0.9% sodium chloride solution without propacetamol and then oral dexibuprofen was administered. We checked the body temperature of all patients at 0.5 h (hr), 1 h, 1.5 h, 2 h, 3 h, 4 h, and 6 h after oral placebo or dexibuprofen had been applied.

**Results:**

A total of 263 patients (125 in the study group) were finally enrolled. The body temperatures of patients in the study group were significantly lower until 2 h after administration (37.73 ± 0.58 vs 38.36 ± 0.69 °C (*p* < 0.001), 37.37 ± 0.53 vs 37.88 ± 0.69 °C (*p* < 0.001), 37.27 ± 0.60 vs 37.62 ± 0.66 °C (*p* < 0.001), 37.25 ± 0.62 vs 37.40 ± 0.60 °C (*p* = 0.0452), at 0.5 h, 1 h, 1.5 h, and 2 h, respectively). The two groups showed no significant differences in terms of the range of body temperature decrease, the Area Under the Curve of body temperature change for antipyretic administration-and-time relationship, the maximum value of body temperature decrease during the 6 h test period, the number of patients whose body temperature normalized (< 37.0 °C), the mean time when first normalization of body temperature, and the development of adverse events including gastrointestinal problem, elevated liver enzyme, and thrombocytopenia.

**Conclusions:**

Intravenous propacetamol may be a safe and effective choice for pediatric URTI patients presenting with fever who are not able to take oral medications or need faster fever control.

**Trial registration:**

CRIS KCT0002888. Date of registration: July 31st, 2013.

## Background

Fever is a common symptom in numerous pediatric diseases including infection and works as a positive response that aids in immune function [1–4]. However, fever confers discomfort, may lead to increased body water loss and dehydration, and may delay overall recovery due to decreased activity and appetite. In such circumstances, antipyretics are used in the pediatric population to alleviate secondary effects of fever like dehydration. Acetaminophen and nonsteroidal anti-inflammatory drugs (NSAIDs) including ibuprofen are commonly used. However, since these drugs are administered via oral route, uses are limited, not being able to be provided for those who cannot take oral medications.

Propacetamol is a prodrug of paracetamol (acetaminophen); 0.5 g of paracetamol can be obtained through plasma esterase-involved hydrolyzation of 1 g of propacetamol [5, 6]. In adult patients, intravenous propacetamol is indicated for fever and acute pain relief. A limited number of previous studies have presented the antipyretic efficacy of intravenous propacetamol in children [7–10]. Furthermore, there have not been previous comparison studies over oral antipyretics and intravenous propacetamol.

Here, we aimed to evaluate and verify the non-inferiority of intravenous propacetamol compared to dexibuprofen in terms of antipyretic efficacy and safety for fever reduction in pediatric upper respiratory tract infection (URTI) patients.

## Methods

### Study design and procedures

This study was a multicenter, randomized, double-blind, comparative, phase 3 clinical trial that was designed to test the antipyretic efficacy of propacetamol (Yungjin Pharm. Co. Ltd., Seoul, Republic of Korea) compared with dexibuprofen (Hanmi Pharm. Co. Ltd., Seoul, Republic of Korea). Subjects from hospitals of The Catholic University of Korea were evaluated for appropriateness for enrollment and were randomized to either the study or control group. The sample size was calculated according to the assumptions stated in the following steps. The level of significance was 0.05, and the power of test was set as 80%. The mean change in body temperature at 6 h after a single dose of 5 mg/kg of dexibuprofen was 0.8 °C with a standard deviation of 1.0 °C. The equivalence margin was − 0.35 with a drop-out rate of 20%.

Study group subjects were administered propacetamol when fever (defined as axillary temperature ≥ 38 °C) developed at a dose of 15 mg/kg in patients weighing < 10 kg and 30 mg/kg in patients weighing ≥10 kg. The dosage of propacetamol was determined according to the previous study [7] which had elucidated the antipyretic effect of intravenous propacetamol, which was administered to children aging from 3 to 12 years at a dose of 30 mg/kg. Because younger and smaller children were included in our study, the dosage of propacetamol for children weighing < 10 kg was determined based on another reference [11]; the propacetamol was mixed with 100 mL of 0.9% sodium chloride solution and given as an intravenous infusion over 30 min. Oral placebo was subsequently administered. The control group subjects were administered intravenous infusion with 100 mL of 0.9% sodium chloride solution without propacetamol for 30 min followed by a single 6 mg/kg dose of oral dexibuprofen. If the subject vomited within 15 min of placebo or dexibuprofen administration, another dose of previously administered oral agent was administered. Body temperature was checked at 0.5 h (hr), 1 h, 1.5 h, 2 h, 3 h, 4 h, and 6 h after placebo or dexibuprofen administration. No further antipyretics and no antibiotics were administered within 6 h of placebo or dexibuprofen administration unless judged necessary by the attending pediatrician.

The study was conducted in accordance with the ethical principles of the Declaration of Helsinki. Written informed consent was obtained from parents or legal guardians and from the child, if possible. The clinical studies were approved by the Korean Food and Drug Administration. The protocol was approved by the Institutional Research Board (IRB) of each institution. Once a patient qualifying the inclusion criteria was enrolled, this patient was prospectively registered in the IRB registry and was then grouped at the ratio of one-to-one into A or B group consecutively by block randomization. The IRB numbers of the participating hospitals are as follows: KC13MDMT0120 at Seoul St. Mary’s Hospital; VC13MDMT0024 at St. Vincent’s Hospital; KMC2015–009 at Hanjin General Hospital; DC14MDMT0006 at Daejeon St. Mary’s Hospital; PS13MDMT0015 at St. Paul’s Hospital; OC13MDMT0025 at Incheon St. Mary’s Hospital; and CR115093 at Yonsei Christian Hospital.

### Inclusion and exclusion criteria

Patients ranging in age from 6 months to 14 years admitted for URTI and presenting with fever (defined as body temperature of the axillar fossa ≥38.0 °C) at the time of admission were included. URTI was diagnosed based on disease history and physical examination carried out by the attending pediatricians. Patients were excluded under the following circumstances: the patient had been administered antipyretics within 4 h prior to admission, a history of febrile crisis within the past 6 months, the presence of severe hematological abnormality, currently receiving treated for or was treated within the past 6 months for nephrologic, hepatologic, pulmonary, endocrine, hematologic, or cardiologic illnesses, neurologic or central nervous system abnormality, diabetes currently not under control, suspected lower respiratory tract infection, severe hemolytic anemia, under maintenance therapy for bronchial asthma, asthma, urticarial, or allergic reaction history when using aspirin or NSAIDs, physical or psychological status deemed inappropriate for a clinical trial, participation in another clinical trial involving other drug(s) within the past 4 weeks, and failure to receive informed consent from the patient or parent.

### Efficacy assessments

The primary efficacy variable was the difference in body temperature reduction at 4 h after antipyretic administration between the study and control groups. The secondary efficacy variables were range of body temperature reduction at 4 h after antipyretic administration, the Area Under the Curve (AUC) of body temperature change until 6 h after antipyretic administration-and-time relationship, the maximum value of body temperature reduction within the 6 h after antipyretic administration, the number of patients whose body temperature normalized (< 37.0 °C) at 6 h after antipyretic administration, and the time point when body temperature first reached< 37.0 °C.

### Safety assessments

Before the administration of antipyretics and at the second visit (3 days after the initial administration), physical examination and laboratory tests with complete blood cell count, blood chemistry analysis, and urinalysis were done. Adverse events were monitored throughout the whole study period and any occurrences were charted.

### Statistical analysis

Test power was set at 80%, and significance level was set at *p* < 0.05. With an expected drop-out rate of 20%, the sample size was calculated to be 161 subjects in each group.

For characteristics analysis, t-tests were used for continuous variables, and Chi-square or Fisher’s exact test were used for categorical variables. For assessing primary efficacy – which is the difference in body temperature reduction at 4 h after antipyretic administration between the study and control groups – propacetamol was considered at least as effective as dexibuprofen if the lower boundary of the 95% confidence interval (CI) for the difference in body temperature reduction (dexibuprofen minus propacetamol) was zero or greater at the equivalence margin of 0.35 °C. Secondary efficacy variables were tested using t-tests, except for the number of patients whose body temperature normalized (< 37.0 °C) at 6 h after antipyretic administration, and the incidence of adverse events during the study period was tested with Chi-square or Fisher’s exact test.

## Results

**Fig. 1:**
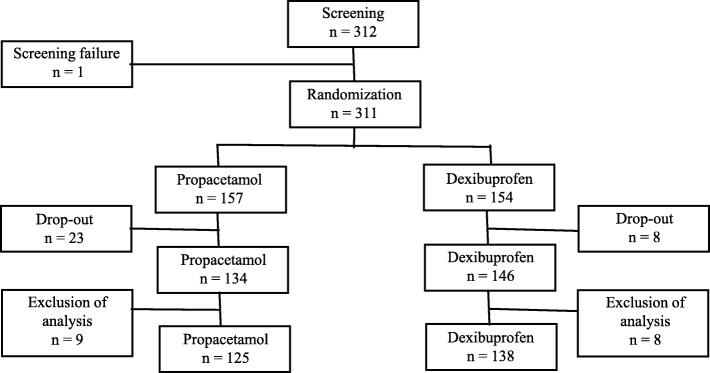
Flowchart comparing patients receiving paracetamol and dexibuprofen in this clinical trial

Three hundred eleven subjects were enrolled during the study period and were randomly assigned to either group (157 in the study group and 154 in the control group). Among them, 23 in the study group and 8 in the control group were excluded due to wanting to drop-out during the study period (12 in the study group and 4 in the control group), withdrawing informed consent (6 in the study group and vs 1 in the control group), receiving prohibited medication during the study period (5 in the study group and vs 3 in the control group). One hundred thirty four subjects in the study group and 146 subjects in the control underwent per protocol analysis, and 17 more subjects were excluded for various reasons (administration of drugs prohibited for concomitant use, withdrawal of parental consent, violation of the time point of body temperature measurement, etc.). The subjects ultimately qualified to be enrolled in our study were selected, and finally 125 subjects in the study group and 138 subjects in the control group were enrolled (Fig. 1). Of the 125 study group subjects, 17 (13.6%) weighed < 10 kg and received 15 mg/kg of propacetamol, and 108 (86.4%) weighed ≥10 kg and received 30 mg/kg of propacetamol. The demographics and basic characteristics were not significantly different between the two groups (Table 1).

**Table 1:** Demographic and clinical characteristics of the study groups

Characteristics	Study groups (*n* = 125)	Control group (*n* = 138)	*p*-value
Gender, male (%)	63 (50.4)	70 (50.7)	0.957
Age (years)	3.0 [0–14.0]	3.0 [0–13.0]	0.730
0.5–1 year (%)	35 (28.0)	41 (29.7)	0.700
2–5 years (%)	65 (52.0)	68 (49.3)	
6–10 years (%)	20 (16.0)	26 (18.8)	
11–14 years (%)	5 (4.0)	3 (2.2)	
Weight (kg)	13.9 [7.4–88.0]	15.0 [7.5–51.0]	0.515
Baseline temperature (°C)	38.6 ± 0.5	38.7 ± 0.5	0.159
Laboratory test results (at admission)			
White blood cell count (× 10^3^/μL)	9.7 [2.7–28.3]	9.6 [1.9–27.7]	0.555
Neutrophil (%)	60.0 [7.7–91.0]	63.7 [16.9–95.0]	0.208
Lymphocyte (%)	29.1 [4.0–86.8]	24.8 [2.0–73.2]	0.134
Platelet (×10^3^/μL)	246.0 [102.0–583.0]	251.0 [91.0–504.0]	0.824
C-reactive protein (mg/μL)	1.68 [0.1–105.1]	2.33 [0.1–139.1]	0.486

Results are presented as median [range] or as mean ± standard deviation or as a percentage (%)

### Efficacy results

The lower boundary of the primary efficacy variable (the difference of body temperature reduction at 4 h after antipyretic administration: dexibuprofen minus propacetamol) was − 0.34, which was within the equivalence margin of 0.35 (Table 2).

**Table 2:** Difference in axillary body temperature reduction at 4 h after antipyretic administration: dexibuprofen minus propacetamol

Efficacy variable	mean ± standard deviation	95% confidence interval	equivalence margin
Dexibuprofen minus propacetamol	−0.13 ± 0.11	(− 0.34, 0.03)	0.35

The area under the curve (AUC) of body temperature change at 6 h after antipyretic administration-and-time relationship did not significantly differ between the two groups. None of the secondary efficacy variables were statistically different between the test and control groups (Table 3).

**Table 3:** Efficacy analysis

Efficacy variable	Study group (*n* = 125)	Control group (*n* = 138)	p-value
AUC of BT change at 6 h after administration-and-time relationship	5.98 ± 3.87	5.78 ± 4.01	0.683
BT reduction at 4 h after administration (°C)	0.97 ± 0.90	1.16 ± 0.92	0.09
Maximum value of BT reduction during the 6 h after administration (°C)	1.63 ± 0.66	1.64 ± 0.70	0.855
Number of patients whose BT normalized (< 37.0 °C) at 6 h after administration, n (%)	26 (20.8)	23 (16.7)	0.390
Time point when BT first reached < 37.0 °C, hour	1.73 ± 1.29	2.13 ± 1.06	0.064

Results are presented as mean ± standard deviation or as a percentage (%)

*BT* Body Temperature

*AUC* Area Under the Curve

**Fig. 2:**
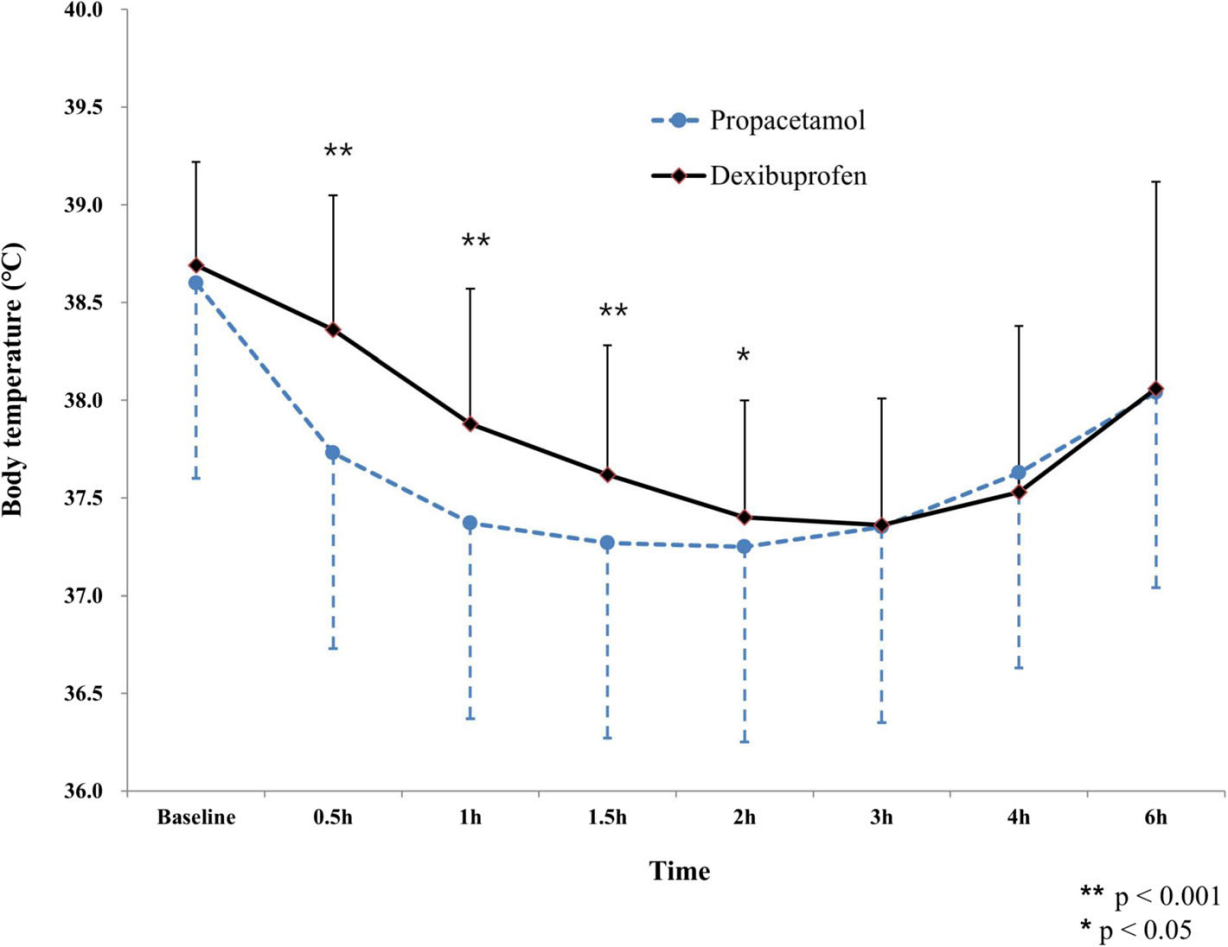
Changes of mean temperature (°C) after the administration h: hour

Body temperatures at 0.5 h, 1 h, 1.5 h, and 2 h after antipyretic administration were significantly lower in the study group (37.73 ± 0.58 °C versus 38.36 ± 0.69 °C, 37.37 ± 0.53 °C versus 37.88 ± 0.69 °C, 37.27 ± 0.60 °C versus 37.62 ± 0.66 °C, and 37.25 ± 0.62 °C versus 37.40 ± 0.60 °C [study vs control group]), while the temperatures at 3, 4, and 6 h after medication administration did not significantly differ. Body temperature < 38 °C was achieved within 0.5 h after administration of propacetamol, while it took approximately 1 h to achieve body temperature < 38 °C after administration of dexibuprofen. For both types of antipyretics, body temperature achieved the lowest value at 2 h after administration (Fig. 2).

### Safety results

A total of 84 adverse events in 64/263 patients were reported. Adverse events included vomiting, diarrhea, abdominal pain, constipation, rash, elevated liver enzyme, and thrombocytopenia. Laboratory adverse events were developed in 21 patients in the study group versus 36 in the control group. AST elevation was found in 8 patients in the study group versus 14 in the control group. ALT elevation was found in 5 patients in the study group versus 9 in the control group. Thrombocytopenia was found in 8 patients in the study group versus 13 in the control group. These laboratory adverse events were assessed as unlikely to be related or unrelated with the type of antipyretics administered. There was no statistically significant difference in adverse event levels between the study group and control group (Table 4). There was no case of study interruption or antipyretic dosage change due to adverse events. There were no serious adverse events in which the patient(s) had been exposed to a danger to life, required a longer hospital stay, or had acquired permanent or major sequalae.

**Table 4:** Number of children with adverse events

	Study group (*n* = 125)	Control group (*n* = 138)	*p*-value
Vomiting	1 (0.8)	4 (2.9)	0.373
Diarrhea	3 (2.4)	7 (5.1)	0.340
Abdominal pain	0 (0)	1 (0.7)	–
Constipation	1 (0.8)	0 (0)	–
Rash	5 (4.0)	5 (3.6)	–
Elevated liver enzyme level			
AST	8 (6.4)	14 (10.1)	0.373
ALT	5 (4.0)	9 (6.5)	0.420
Thrombocytopenia	8 (6.4)	13 (9.4)	0.495

Results are presented as a percentage (%)

*AST* aspartate aminotransferase

*ALT* alanine aminotransferase

## Discussion

Based on our study results, the antipyretic effect of intravenous propacetamol compared to dexibuprofen used in pediatric URTI patients presenting with fever was similar. In addition, concerning safety issues, intravenous propacetamol was tolerable based on our data analysis.

Dexibuprofen and acetaminophen are the two most widely used antipyretic drugs in the pediatric population. The former is an enantiomer of racemic ibuprofen, an effective and tolerable antipyretic and analgesic drug for pediatric use [12–14], and an equal effect at a lower dose than ibuprofen has been shown in previous studies [15–17], some including pediatric upper respiratory tract infection (URTI) patients presenting with fever [18, 19]. Acetaminophen is another popular choice of pediatric antipyretic drug, which is generally administered via oral route. However, a rectal route may be used in cases when the oral route is not tolerable, such as when the patient is vomiting, in respiratory distress, or has decreased mental status. In such a case, its bioavailability is substantially reduced (54% lower than that for the oral route), making it difficult to quantify the targeted drug concentration [20]. In such circumstances, intravenous antipyretic like propacetamol (a prodrug of acetaminophen as previously mentioned) would be a preferred choice.

In addition, if prompt alleviation of fever is warranted in severe pyrexia, intravenous antipyretics may be indicated [21]. In our study, the body temperature during the first 2 h after intravenous propacetamol administration was significantly lower than that after dexibuprofen administration. While intravenous drug concentrations reach maximum levels within 40 min when propacetamol is intravenously administered [22], it takes more than 2 h for dexibuprofen to reach its maximum concentration after oral administration [15]. This difference may have influenced our results concerning the superior antipyretic effect of intravenous propacetamol within the first 2 h after administration. Such rapid antipyretic effect of propacetamol may be promising in preventing recurrent febrile seizures, because approximately half of the recurrent seizure events are encountered in the first 2 h after a second fever episode [23]. Beyond 3 h after antipyretics administration, the BT change between the two groups did not differ significantly. This may be associated with the half-life of each antipyretic drug (1.8–3.5 h for dexibuprofen and 2.1–4.8 h for propacetamol) [24, 25]. Once the plasma concentration of the drug is reduced, the antipyretic effect would be diminished and thus lead to sequential rise in BT, minimizing the significant difference of BT between the two groups in the later hours after antipyretic administration.

Furthermore, propacetamol has another advantage over NSAIDs in that it interferes less with platelet functions. In previous literature, propacetamol was shown to be related with reversible platelet dysfunction but at a lesser extent compared to ketorolac [26]. Further, in more recent reports, paracetamol – the hydrolyzed product of propacetamol – has been studied for its efficacy and safety in preterm infants for treatment of patent ductus arteriosus, and has shown less adverse effects concerning platelet function [27]. Therefore, propacetamol may be safely used in patients with hemorrhage risks or underlying hematologic diseases. Also, the safety profile of propacetamol is known to be superior to that of NSAIDs for use in patients with a history of peptic ulcers or asthma [28].

Meanwhile, the recommended dosage of acetaminophen varies depending on the age or weight of the patient. For example, Fusco et al. [29] administered 7.5 mg/kg, 10 mg/kg, and 15 mg/kg of acetaminophen to children < 3 months, ≥ 3 months and < 24 months, ≥ 24 months old, respectively. In our study, we administered 15 mg/kg of propacetamol (7.5 mg/kg of acetaminophen) in patients weighing < 10 kg and 30 ml/kg of propacetamol (15 mg/kg of acetaminophen) in patients weighing ≥10 kg. Complying with this set criteria, the actual dosage administered was equal to or less than previously known dosages (provided that a child reaches 10 kg at 12 months of age), but the antipyretic effect was satisfactory and the safety profiles were acceptable.

The adverse effect(s) of a drug is also an issue to take a cautious notice in. Pain at the injection site is a typical adverse event of intravenous propacetamol administration, which was shown to reach 10.0% in a previous publication by Walson et al. [7]. However, Walson and colleagues showed that pain at the injection site was 9.5% even in the placebo group. Such pain can be alleviated by slow infusion of the drug [5]. In this study, we diluted propacetamol in 100 ml of 0.9% sodium chloride solution and slowly intravenously infused the drug for 30 min, and pain at the injection site was not reported.

This study is limited in that intention-to-treat analysis was not done, which necessitates complements in future researches. Also, future studies are warranted to evaluate the antipyretic efficacy and safety of intravenous propacetamol involving more various disease entities. Furthermore, supplemental researches over the combination or alternation therapy of propacetamol and other po antipyretic (i.e, NSAIDs) are required.

## Conclusion

We were able to verify the antipyretic efficacy and safety of intravenous propacetamol in febrile pediatric URTI patients. Intravenous propacetamol may be used effectively in patients for whom oral antipyretics cannot be administered or a prompt antipyretic is warranted.

## Abbreviations

NSAIDs: Nonsteroidal anti-inflammatory drugs
URTI: Upper respiratory tract infection
